# Editorial: Genetic and molecular mechanisms of healthy aging and age-related diseases

**DOI:** 10.3389/fgene.2024.1509961

**Published:** 2024-10-25

**Authors:** Yunpeng Xu, Jiang-An Yin, Zhiyong Shao

**Affiliations:** ^1^ Department of Molecular Biology and Biochemistry, Rutgers University, Piscataway, NJ, United States; ^2^ Institute of Neuropathology, University of Zurich, Zurich, Switzerland; ^3^ State Key Laboratory of Medical Neurobiology and MOE Frontiers Center for Brain Science, Department of Neurosurgery, Institutes of Brain Science, Zhongshan Hospital, Fudan University, Shanghai, China

**Keywords:** aging, genetic, molecular, age-related diseases, lifespan

Aging is an intricate biological process involving gradual changes at the cellular, molecular, and genetic levels (Lopez-Otin et al., 2023; Herndon et al., 2002). These changes lead to the decline of physiological functions and increase vulnerability to age-related diseases. Unlike lifespan, which is measured by a single metric—how long a species can survive—natural aging is a complex process that affects most, if not all, tissues, and organs. It remains largely unknown whether different tissues and organs age through common mechanisms and whether these mechanisms are conserved across species. Recent studies have explored the molecular pathways behind aging, focusing on key systems such as the respiratory tract, muscles, immune system, and stress responses. Understanding these mechanisms can provide insights for developing therapies to promote healthspan and address age-related conditions.

## Airway aging and immune dysfunction

The respiratory system is one of the primary areas where aging-related decline is evident. Cellular and functional changes in the airway, particularly in the trachea and bronchus, contribute to diseases such as chronic obstructive pulmonary disease (COPD) and asthma. The trachea and bronchus consist of multiple cell types, but which cells are most susceptible to aging remains unknown. A detailed landscape of gene expression changes at the cell-type level in aging airways is urgently needed. A recent study by Liu et al., through RNA sequencing and cell-type specific analyses, reveals that the proportion of basal cells, which play a critical role in airway regeneration, decreases significantly with age. This reduction is associated with a weakened ability for tissue repair and regeneration. Moreover, these age-related changes are not limited to the structural components of the airway. Immune regulatory pathways, including TNF and NF-kappa B signaling, become increasingly dysregulated with age. This dysregulation leads to heightened inflammation, which can exacerbate chronic respiratory diseases. While these findings are intriguing, they are limited to females, and confirmatory studies are needed to determine if they are consistent in males. Further, it is worthy to validate the relationship between basal cell loss and immune pathway dysfunction. Specifically, it is important to determine whether one drives the other, or if they occur concomitantly during airway aging, and which specific airway aging character is regulated by one or both of them.

## Sarcopenia and mitochondrial dysfunction

Sarcopenia is characterized by the loss of muscle mass and strength and affects the mobility and overall life quality of the aging population. Cuproptosis has been implicated in sarcopenia, and identification and validation of cuproptosis-related genes in sarcopenia will offer new insights and could provide therapeutic targets. A study by Zhu et al., through comprehensive bioinformatic analyses of gene expression profiles from muscle biopsies of individuals with sarcopenia and healthy controls, identifies some cuproptosis-related genes including PDHA1, DLAT, PDHB, and NDUFC1 as key biomarkers for diagnosing and potentially treating sarcopenia. These genes are closely linked to mitochondrial energy metabolism, which is crucial for maintaining muscle function. Mitochondrial dysfunction is increasingly recognized as a driving factor in sarcopenia, leading to impaired muscle repair and regeneration. Interestingly, metformin, a drug traditionally used to treat diabetes, has shown promise as a potential therapeutic agent for sarcopenia by targeting mitochondrial pathways. This research underscores the importance of mitochondrial health in combating age-related degenerative diseases and opens a new avenue for sarcopenia treatments.

## TGF-β signaling in aging and immunosenescence: lessons from *Caenorhabditis elegans*

TGF-β, a member of the superfamily of signaling molecules, is pivotal in various biological processes, including development, homeostasis, and pathogenesis. Studies utilizing the model organism *C. elegans* have significantly advanced our understanding of the role and underlying mechanisms of TGF-β signaling (Fan et al., 2022). A review by Yamamoto and Savage-Dunn summarizes recent research on TGF-β pathways in aging and immunity. As organisms age, they experience a decline in physiological functions and an increase in susceptibility to diseases. TGF-β signaling pathway members including TGF-β/Activin and BMP (Bone Morphogenetic Protein), have been identified as key regulators in this process. TGF-β signaling not only influences longevity but also has profound implications for immune system health. This dual role underscores the interconnectedness of aging and immune responses, a phenomenon known as immunosenescence. Research in *C. elegans* has revealed non-canonical aspects of TGF-β signaling that may be relevant for mammalian systems. For instance, a study highlighted the presence of TGF-β signaling and insulin/IGF-1 signaling (IIS) as fundamental pathways associated with healthy aging, particularly in centenarians. The findings suggest that understanding TGF-β′s regulatory mechanisms can lead to insights that may enhance our knowledge of aging and improve health outcomes across species. Overall, the insights gained from TGF-β signaling in *C. elegans* may be applicable to broader biological contexts, particularly regarding aging and immunity.

## Integrated stress response and aging

The integrated stress response (ISR) plays a pivotal role in managing cellular stress through modulating protein synthesis, particularly in response to nutrient limitation and endoplasmic reticulum (ER) stress. Dietary restriction (DR) is a promising way of extending lifespan and preserving healthspan in various species. It is unclear whether ISR is involved in protein synthesis and lifespan extension in model organisms under DR. A study by Ma et al. discovered that DR in *C. elegans* reduces mRNA translation, activates the ISR, and extends the lifespan of the animals. Interestingly, they found that reduced mRNA translation and extended lifespan occur independently of eIF-2α phosphorylation, a key feature of ISR. However, eIF-2α phosphorylation remains crucial for managing ER stress and ensuring normal survival in these animals. This finding suggests that ISR regulates ER stress and longevity via distinct mechanisms. The other regulatory mechanisms such as nutrient sensing pathways and the unidentified substrate(s) of kinases of GCN2 and PEK-1 implied in the study may be more directly involved in promoting longevity. Understanding how different stress response pathways interact during aging could reveal new targets for extending lifespan and improving stress resilience in older individuals.

## Oxidative stress and genetic susceptibility

Oxidative stress, characterized by an imbalance between reactive oxygen species (ROS) production and antioxidant defenses, is a key factor in aging and age-related diseases. Genetic variations in pro-oxidant and antioxidant genes significantly influence an individual’s ability to manage oxidative stress. The review by Krishnamurthy et al. systematically summarizes the single nucleotide polymorphisms occurring in oxidative stress-related prooxidant and antioxidant genes in human populations. They highlight polymorphic variations impacting the expression and activity of the encoded proteins may contribute to the redox imbalance in affected individuals. These genetic variations suggest the therapeutic potential for personalized medicine. By identifying those at high risk of oxidative damage due to genetic factors, healthcare providers can implement targeted strategies to mitigate oxidative stress and improve longevity and other related health issues.

## Conclusion: toward a comprehensive understanding of aging mechanisms

These studies and reviews, focusing on key systems such as the respiratory tract, muscles, immune function, and stress responses, represent the latest advancements in the molecular mechanisms underlying aging. As summarized above, each of these systems contributes to the aging process through defined pathways that regulate cell renewal, immune response, and oxidative balance, indicating the complex feature of the aging process of a defined functional aspect. Thus, targeting specific roots causing the dysfunction—such as mitochondrial metabolism, immune regulation, and oxidative stress—may provide a strategy for treating specific malfunctions. Although it is still unclear whether a global master target for a broad spectrum of aging processes exists, we believe that as research continues to advance, the integration of genetic insights, bioinformatics, and therapeutic interventions will improve healthspan and reduce the burden of age-related diseases.
